# Folate Decorated Nanomicelles Loaded with a Potent Curcumin Analogue for Targeting Retinoblastoma

**DOI:** 10.3390/pharmaceutics9020015

**Published:** 2017-04-18

**Authors:** Hashem Alsaab, Rami M. Alzhrani, Prashant Kesharwani, Samaresh Sau, Sai HS. Boddu, Arun K. Iyer

**Affiliations:** 1 Pharmaceutical Sciences, Eugene Applebaum College of Pharmacy and Health Sciences, 259 Mack Ave., Wayne State University, Detroit, MI 48201, USA; hashem.alsaab@wayne.edu (H.A.); rami.alzhrani@wayne.edu (R.M.A.); prashantdops@gmail.com (P.K.); samaresh.sau@wayne.edu (S.S.); 2 Department of Pharmaceutics and Pharmaceutical Technology, Taif University, Taif, 26571, Saudi Arabia; 3 Pharmaceutics Division, CSIR-Central Drug Research Institute, Lucknow 226031, India; 4 Department of Pharmacy Practice, The University of Toledo, Health Science Campus, 3000 Arlington Ave., Toledo, OH 43614, USA; sboddu@utnet.utoledo.edu; 5 Molecular Therapeutics Program, Barbara Ann Karmanos Cancer Institute, Wayne State University, School of Medicine, Detroit, MI 48201, USA

**Keywords:** retinoblastoma, Y-79, WERI-RB, folate-receptor targeting, nanomicelles, 3,4-difluorobenzylidene curcumin (CDF), fluorocurcumin, targeted drug delivery

## Abstract

The aim of this study was to develop a novel folate receptor-targeted drug delivery system for retinoblastoma cells using a promising anticancer agent, curcumin-difluorinated (CDF), loaded in polymeric micelles. Folic acid was used as a targeting moiety to enhance the targeting and bioavailability of CDF. For this purpose, amphiphilic poly(styrene-*co*-maleic acid)-conjugated-folic acid (SMA-FA) was synthesized and utilized to improve the aqueous solubility of a highly hydrophobic, but very potent anticancer compound, CDF, and its targeted delivery to folate overexpressing cancers. The SMA-FA conjugate was first synthesized and characterized by ^1^H NMR, FTIR and DSC. Furthermore, the chromatographic condition (HPLC) for estimating CDF was determined and validated. The formulation was optimized to achieve maximum entrapment of CDF. The particle size of the micelles was measured and confirmed by dynamic light scattering (DLS) and transmission electron microscopy (TEM). Cytotoxicity studies were conducted on (Y-79 and WERI-RB) retinoblastoma cells. Results showed that the solubility of CDF could be increased with the newly-synthesized polymer, and the entrapment efficiency was >85%. The drug-loaded nanomicelles exhibited an appropriate size of <200 nm and a narrow size distribution. The formulation did not show any adverse cytotoxicity on a human retinal pigment epithelial cell (ARPE-19), indicating its safety. However, it showed significant cell killing activity in both Y-79 and WERI-RB retinoblastoma cell lines, indicating its potency in killing cancer cells. In conclusion, the folic acid-conjugated SMA loaded with CDF showed promising potential with high safety and pronounced anticancer activity on the tested retinoblastoma cell lines. The newly-formulated targeted nanomicelles thus could be a viable option as an alternative approach to current retinoblastoma therapies.

## 1. Introduction

Retinoblastoma is a rare intraocular tumor that is caused by a mutation in the retinoblastoma-associated protein (RB1 gene), which acts as a tumor suppressor [1]. RB affects all genders and races equally and causes 1% of cancer death in children and 5% of blindness [2]. According to American Cancer Society 2015 statistics, about 200–300 children have been diagnosed with RB annually. Furthermore, three out of four of those children are suffering from one eye tumor. Untreated RB is dangerous and fatal due to propagation of the disease within two years. The ultimate goal of treatment increases patient survival followed by vision protection [2]. Even though the cure rate of RB is about 95%, the secondary neoplasms that occur after conventional RB treatment play a significant role in reducing survival rate due to a mutation in the RB1 gene [3,4]. RB1 has a crucial role in maintaining the stability of chromosomes. RB1 contributes to regulating epigenetic processes such as DNA methylation and histone modification; thus, RB1 inactivation leads to retinoblastoma [5].

A recently-synthesized curcumin analog, called 3,4-difluorobenzylidene curcumin (CDF), also known as difluorinated curcumin, is a synthetic derivative of curcumin and has shown a 16-fold increased half-life and high anticancer activity compared to curcumin when tested on pancreatic cancer cells [6]. Curcumin′s poor bioavailability and its rapid inactivation via glucuronidation are some of the major drawbacks that hinder its therapeutic activity [7]. On the other hand, the fluorocurcumin analog has shown higher metabolic stability than curcumin [8,9]. Moreover, Kanwar et al. have shown that CDF poses greater growth-inhibitory properties compared to curcumin. Furthermore, Kanwar et al. revealed that CDF could inhibit ATP-binding cassette sub-family G member 2 (ABCG2), which plays a major role in chemotherapeutic resistance via activation of efflux activity [10]. One of the major drawbacks of conventional chemotherapeutic agents in RB is the difficulty in the retinal uptake of these agents. Therefore, a high concentration of chemotherapeutic agents is needed, which usually leads to toxicity to normal cells [11]. Exposure of RB to chemotherapeutic agents results in overexpression of the multidrug resistance (MDR) gene that facilitates the efflux of chemotherapeutic agents via overexpression of efflux pumps (P-glycoprotein (P-gp), multidrug resistance protein (MRP) and breast cancer resistance protein (BCRP)) [12]. Furthermore, MDR gene overexpression plays a major role in developing resistance to diverse structural and functionally-variant chemotherapeutic agents [13,14]. Moreover, conventional chemotherapeutic agents cannot differentiate between healthy and cancer cells, eventually resulting in unwanted side effects [15]. Therefore, developing a drug delivery system that can target cancer cells, increase its intracellular uptake and minimize side effects is one of our study goals.

Drug targeting via nutrient transporters/receptors such as amino acids, peptides and folate plays a crucial role in facilitating drug uptake into target cells [16]. Folic acid is an essential cell vitamin that can target overexpressed folate receptors found on the membrane of RB cells, as well as other cancer cells. Folic acid expression, on the other hand, is relatively low or sparse in normal tissues and cells. Therefore, attachment of folic acid on the surface of drug delivery systems, such as liposomes, polymeric micelles and nanoparticles, increases the targeting of these systems to folate-overexpressing cancer cells [17,18].

**Figure 1:**
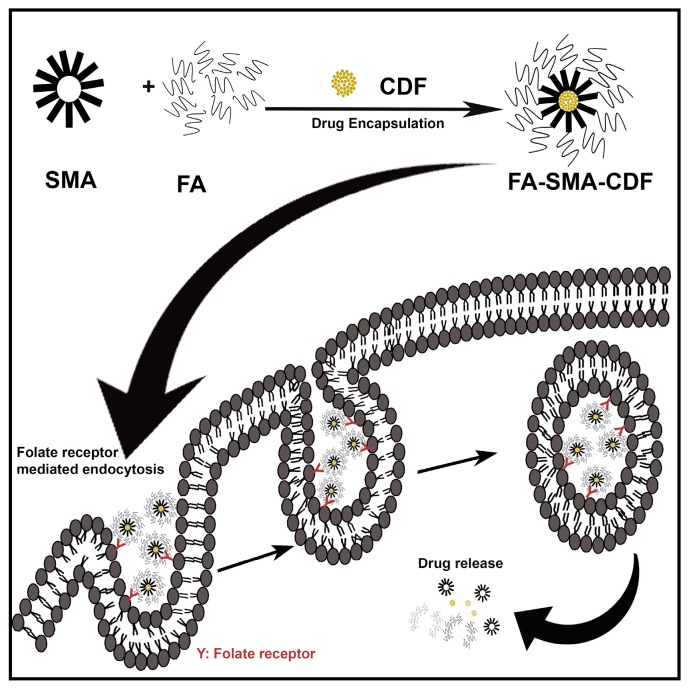
Accumulation of targeted formulation (synthesized amphiphilic poly(styrene-*co*-maleic acid)-conjugated-folic acid (SMA-FA)-CDF) at the tumor via folate receptor-mediated endocytosis due to the specific binding of FA to folate receptors overexpressed on retinoblastoma cells (Y-79).

In this study, we engineered a novel folate receptor targeting formulation for RB. Earlier studies have proven the presence of the folate receptor on the RB (Y-79) cell line; thus, Y-79 cells will serve as a favorable cell line for in vitro testing [19]. We have successfully synthesized amphiphilic poly(styrene-*co*-maleic acid)-conjugated-folic acid (SMA-FA)-CDF micelles for targeting the RB folate receptor in which the micelles would be internalized to RB cells via endocytosis, resulting in better anticancer activity as shown in Figure 1.

## 2. Methods

### 2.1. Materials

3,4-Difluorobenzylidene curcumin (CDF) was synthesized as described earlier [20,21]. Poly (styrene-co-maleic anhydride) (average MWt 1600), *N*-(3-(dimethylamino) propyl)-*N*-ethylcarbodiimide hydrochloride (EDC) and 3-[4,5dimethylthiazol-2-yl]-2,5diphenyltetrazolium bromide (MTT) were provided by Sigma-Aldrich (St. Louis, MO, USA). FA was purchased from Fisher Scientific, Waltham, MA, USA. All other chemicals were of reagent grade and used without any modification. For the cell culture, Y79 and WERI-RB1 were procured from ATCC^®^ (Manassas, VA, USA). Retinoblastoma Y79 cells were maintained in Roswell Park Memorial Institute medium (RPMI 1640) with 20% fetal calf serum, 0.37% sodium bicarbonate, 0.058% l-glutamine, 10 mM HEPES and 100 μg/mL gentamicin. WERI-RB1 cells were maintained in Dulbecco’s Modified Eagle’s Medium (DMEM) with 10% calf serum, 1× MEM nonessential amino acids (GIBCO, Grand Island, NY, USA), 1× MEM vitamins (GIBCO, Grand Island, NY, USA), 0.37% sodium bicarbonate, 0.058% l-glutamine and 100 μg/mL gentamicin.

### 2.2. Synthesis of Folate-Conjugated SMA Copolymer

FA-SMA copolymer was synthesized using the previously-modified method [22]. Firstly, FA solution was prepared by solubilizing known amounts of FA in sodium bicarbonate buffer and magnetically stirring for 6 h keeping the pH of 8.9. In another container, activation of SMA polymer was made by 1-Ethyl-3-(3-dimethylaminopropyl)carbodiimide (EDC) and *N*-hydroxysuccinimide (NHS). After 3 h at room temperature, the reaction was completed. Then, the activated SMA solution was added dropwise to the basic FA solution under continuous stirring overnight at room temperature to synthesize the FA-SMA conjugate. Finally, purification of the FA-SMA conjugate was made by using Millipore tangential flow filtration (TFF) (Millipore, Milford, MA, USA) by ultrafiltration. The final conjugate was lyophilized, characterized and stored in a freezer until further use.

### 2.3. Fabrication FA-SMA-CDF Nanomicelles

FA-SMA-CDF nanomicelles were formulated according to a previously-reported method by our group with some modification [20,21]. Briefly, a known amount of the synthesized FA-SMA conjugate was dissolved in DI water at RT under magnetic stirring, and the pH was adjusted to 5.0. CDF was dissolved in an appropriate quantity of DMSO and added dropwise to the FA-SMA polymer solution. After that, the self-assembly of the hydrophobic parts in CDF and styrene component of SMA led to the formation of nanomicelles. At the same time, EDC was added and stirred in the dark for 30 min to prevent CDF light exposure and the pH kept at 5.0. Then, the slow addition of 1 M NaOH was done to increase the pH to 10.0 till the solution become clear. At the end, the pH was readjusted to 7.4 using 0.1 M HCl and dialyzed overnight using a dialysis membrane (molecular weight cutoff 3.5 kDa, Spectrapor, Spectrum Laboratories, SD, Rancho Dominquuez, CA, USA) against DI water to remove free drug (CDF) and then lyophilized (Eyela Inc., Tokyo, Japan) to obtain the final product.

### 2.4. Characterization of FA-SMACDF Nanomicelles

The structure of FA-SMA was confirmed by ^1^H NMR spectroscopy in d-DMSO. Chemical shifts (d) were expressed in parts per million (ppm) relative to the NMR solvent signal (d-DMSO) using tetramethyl silane as an internal standard. The amount of folic acid conjugated to SMA was determined by a calibration curve of folic acid generated in DMSO at 340 nm. FTIR spectra of the pure CDF, drug-loaded nanomicelles, blank nanomicelles and the physical mixture of FA-SMA were taken using an FTS 4000 FTIR spectrometer (Varian Excalibur Series UMA 600 FTIR, Digilab, Marlborough, MA, USA) equipped with a germanium crystal. A resolution of 16 cm^–1^ was used, and 16 scans were co-added for each spectrum in the range of 500 to 4000 cm^–1^.

### 2.5. Differential Scanning Calorimetry

DSC analysis was carried out for CDF, blank formulation and drug-loaded nanomicelles to examine the change in the rate of heat absorbed by CDF after dissolving in nanomicelles. All samples (5–10 mg) were sealed and placed in aluminum crucibles using the Mettler MT 5 microbalance (Mettler Toledo, Columbus, OH, USA). DSC studies happened at a 10 °C/min heating rate over a wide range (10 to 300 °C) using a DSC 822^e^ Mettler Toledo instrument (Mettler Toledo GmbH, Schwerzenbach, CH, USA) fitted with a TSO801RO sample robot and a TSO800GCI gas control attached to a nitrogen gas cylinder. Star e software V8.10 was used to record the scans. Nitrogen gas was purged at a rate of 10 mL/min.

### 2.6. Particle Size and Morphology

Nanomicelles were characterized for size using a Beckman Coulter Delsa Nano C DLS Particle analyzer (Beckman Coulter, Inc., Fullerton, CA, USA) equipped with a 658-nm He-Ne laser. Polydispersity index (PI) values were also measured. Furthermore, nanomicelles were further characterized for surface morphology by transmission electron microscopy (TEM). TEM (HITACHI HD-2300 A, Ultra-thin Film Evaluation System, Hitachi High Technologies America, Pleasanton, CA, USA) was used in evaluating the morphology of nanomicelles. Samples were prepared by placing a small drop of the formulation on a copper grid. Then, the samples were viewed using TEM.

### 2.7. HPLC Chromatographic Conditions

A high-performance liquid chromatography (HPLC) method was developed and validated for drug content determination. Samples were analyzed using a Waters Alliance e2695 separation module (Milford, MA, USA) equipped with a 2998 PDA detector. A Waters Symmetry^®^ (Milford, MA, USA) C18 column 5 µm (4.6 mm × 250 mm) was used with a mobile phase composed of a binary composition (A:B) (70:30) pumped at a flow rate of 1 mL/min. The mobile phase (A) was 0.45% formic acid in DI water. The mobile phase (B) was methanol. The retention time for CDF (*λ*_max_ = 447 nm) was found to be 9.786 min. CDF stock solution (1 mg/mL) was used in creating calibration curve standards. Each standard concentration was analyzed in triplicate. A calibration curve was developed by plotting the average area against the amount of drug (ng) to determine the drug content. A straight line (*y* = 5140.1*x* – 18319) was obtained with a correlation coefficient (*r*^2^) value of 0.9998, and the standard curve region was from 0.390 to 50 µg/mL. The HPLC method was validated as per regulatory requirements [22]. The percentage recovery of CDF ranged from 97.25% to 100.33%. The intra- and inter-assay precisions of CDF were satisfactory; the RSD was less than 2%. The intra-day precision (measured by %RSD) was found to be in the range of 0.08% to 0.53%. The limits of detection of CDF were found to be 91.1 and 276.2 ng/mL, respectively.

### 2.8. Drug Encapsulation Efficiency

Free drug (non-incorporated in the FA-SMA) was separated by ultrafiltration centrifugation technique. Briefly, 1 mL of CDF and FA-SMA-CDF solution was placed in the upper chamber of a centrifuge tube matched with an ultrafilter and centrifuged for 15 min at 4000 rpm. The total drug content in CDF nano-formulation was determined as follows: aliquots of 1 mL of the formulation dispersion were diluted appropriately by ethanol to dissolve the FA-SMA ingredient, and the resulting suspension was then filtrated through 0.45-µm membrane filters. The filtered solution was analyzed by HPLC. The encapsulation efficiency (EE) and drug loading content (DLC) were calculated by the following equations:

(1) $$\mathrm{Drug}\;\mathrm{loading}\;\mathrm{content}\;\left(\mathrm{DLC}\right)=\frac{\begin{array}{c} \mathrm{weight}\;\mathrm{of}\;\mathrm{CDF}\\ \mathrm{encapsulated}\;\mathrm{in}\;\mathrm{micelles} \end{array}}{\begin{array}{c} \mathrm{Total}\;\mathrm{weight}\;\mathrm{of}\;\mathrm{CDF}\\ \mathrm{loaded}\;\mathrm{in}\;\mathrm{micelles} \end{array}}\times 100$$

(2) $$\mathrm{Encapsulation}\;\mathrm{Effeciency}\;\left(\mathrm{EE}\right)=\frac{\begin{array}{c} \mathrm{Mass}\;\mathrm{of}\;\mathrm{CDF}\\ \mathrm{encapsulated}\;\mathrm{in}\;\mathrm{micelles} \end{array}}{\begin{array}{c} \mathrm{Total}\;\mathrm{mass}\;\mathrm{of}\;\mathrm{CDF}\\ \mathrm{initially}\;\mathrm{loaded}\;\mathrm{in}\;\mathrm{micelles} \end{array}}\times 100$$

### 2.9. Stability Indication Assay

CDF was exposed to different temperatures to investigate the formulation stability further. These conditions included heating at 4, 25 and 35 °C. Samples were later analyzed to quantify the drug percentage, and the recovery percentage was also calculated.

### 2.10. Cell Viability Study on ARPE-19, Y-79 and WERI-RB1

Cell viability was assessed in ARPE-19 using the 3-(4,5-dimethyl-2- thiazolyl)-2,5-diphenyl-2H-tetrazolium bromide (MTT) assay. Briefly, cells were seeded in 96-well plates at a density of 10,000 cells/well and incubated for 24 h in these environments (37 °C in a 5% CO_2_) to enhance cell attachment. Various concentrations of CDF, FA-SMA and FA-SMA-CDF (0.1, 0.25, 0.5 and 0.75 µM) were used to test cells’ cytotoxicity. After 24 h, the medium was aspirated, and the cells were incubated with MTT reagent for 3 h in 5% CO_2_ at 37 °C. The yellow medium was aspirated, and 200 µL of DMSO were added to each well to allow the dissolution of the formazan salt. The absorbance was measured at 570 nm using a spectrophotometer (H^1^ Synergy). Furthermore, human RB cell lines Y-79 and WERI-RB1 were obtained from the American Type Culture Collections. Further cytotoxicity studies were also performed on Y-79 cells. Y-79 cells were incubated in 75-cm^2^ tissue culture flasks as a suspension in RPMI-1640 medium supplemented with 15% non-heat-inactivated fetal bovine serum, 1 mM glutamine, penicillin (100 U/mL) and streptomycin (100 mg/mL). Moreover, the same MTT assay procedures were done for both retinoblastoma cell lines.

### 2.11. Statistical Analysis

All the experiments were carried out in triplicate. Values are expressed as the mean ± standard deviation (SD). The statistical analysis of the data was performed using a Student’s *t*-test, and *p*-values <0.05 were considered statistically significant.

## 3. Results

**Figure 2:**
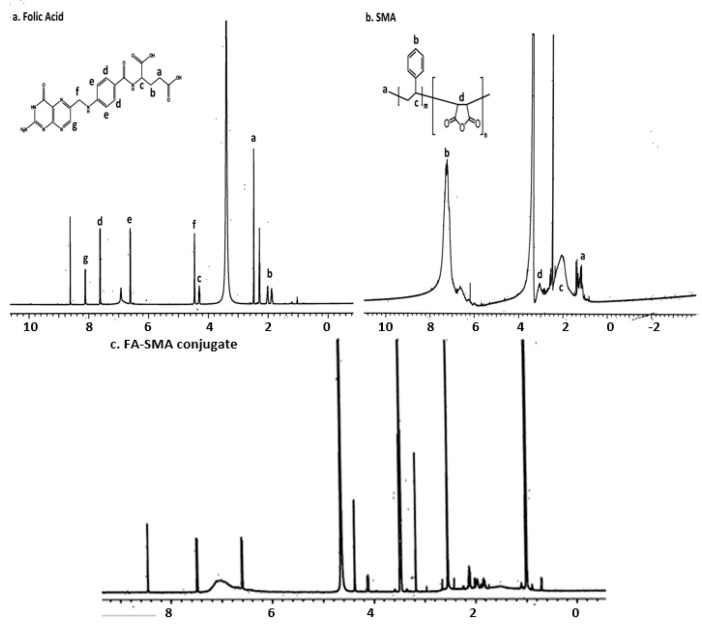

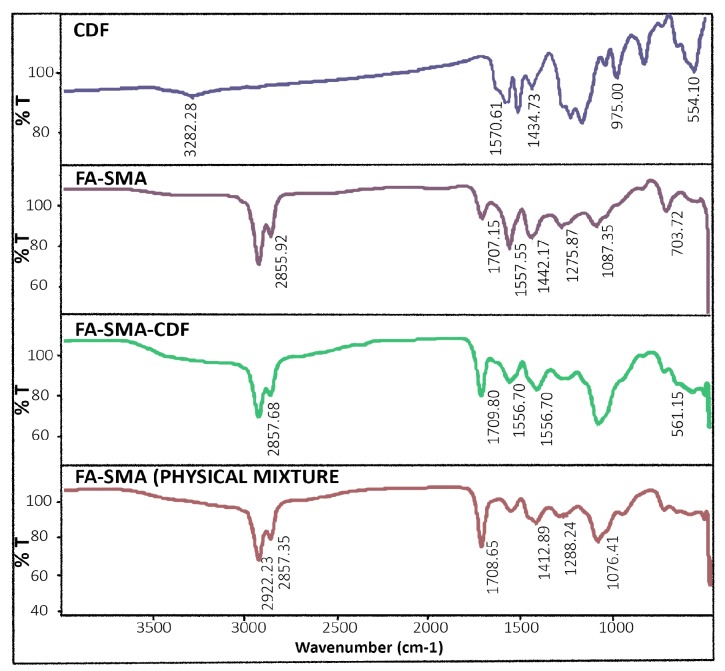
**Up**: ^1^H NMR was performed for (a) folic acid, (b) FA-SMA and FA-SMA-CDF; **Bottom**: FTIR and characterization of CDF, FA, SMA and the FA-SMA conjugate.

**Figure 3:**
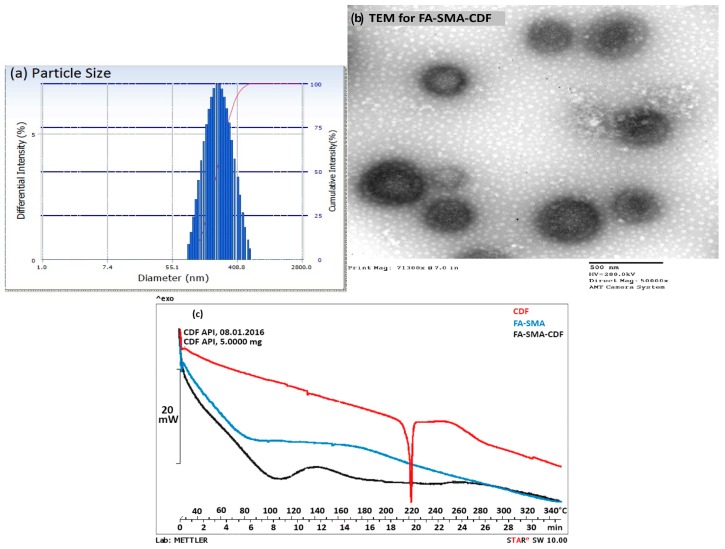
(**a**) Particle size characterization of SMA-CDF and FA-SMA-CDF using the dynamic light scattering (DLS) technique; (**b**) surface morphology of SMA-CDF and FA-SMA-CDF by transmission electron microscopy (TEM); (**c**) differential scanning calorimetry (DSC) of pure drug (CDF), FA-SMA, and FA-SMA-CDF nanoformulations.

We first synthesized a water-soluble copolymer, folic acid conjugated to styrene maleic acid (FA-SMA), and structurally characterized it by ^1^H NMR and FTIR spectroscopy according to a previously-published method with some modifications [20]. Figure 2a demonstrates various proton peaks associated with FA, SMA and FA-SMA conjugate polymer, indicating the formation of the co-polymer. Small peaks at 6.6, 7.6 and 8.7 ppm indicate that protons are associated with folate. The characteristic aromatic peaks of the styrene subunits (SMA) at about 6.2 to 7.2 ppm further confirmed the presence of SMA components in FA-SMA conjugates. Additionally, the FTIR spectrum of FA-SMA revealed a unique characteristic peak at 1707, and 1557 cm^–1^ has confirmed the formation of an amide bond (Figure 2b). Table 1 shows the effect of particle size, polydispersity and encapsulation efficiency. Based on size, polydispersity and entrapment efficiency, FA-SMA was found to be suitable for the preparation of CDF for ophthalmic application. Dynamic light scattering (DLS) was utilized for the measurement of the particle size of nanomicelles. The obtained particle size of the SMA-CDF nanomicelles was 183.4 ± 3.28 nm. However, the particle size of the FA-SMA-CDF nanomicelles was 191.3 ± 2.92 nm (Figure 3a). The nanoformulation was further characterized for morphology using transmission electron microscopy (TEM). The spherical morphology of FA-SMA-CDF was confirmed by TEM studies (Figure 3b). The spherically-shaped nanoparticles of FA-SMA-CDF corroborated the results obtained by DLS. The loading of the drug in nanomicelles was estimated by HPLC. The loading of CDF in SMA-CDF and FA-SMA-CDF was 17.48% ± 4.98% and 12.14% ± 2.23% *w*/*w*, respectively. Furthermore, differential scanning calorimetry (DSC) analysis was carried out for the pure drug, a carrier without the drug and the drug-loaded nanoformulation to examine the enthalpy change in CDF after loading in the nanomicellar formulation. DSC thermograms of pure CDF and the other samples are illustrated in (Figure 3c). CDF exhibited a sharp endothermic peak at around 220 °C, which corresponds to its melting point. The thermograms of the blank nanoformulation (FA-SMA) showed a broad endothermic peak at 110 °C due to the release of water molecules/moisture present in the nanoformulations. Drug-loaded nanomicelles exhibited a small shift in endothermic peaks from 110 to around 115 °C, and the CDF peak was completely absent. This indicated the absence of any undissolved CDF in the nanomicellar formulation, and the drug is completely in its solubilized form.

**Figure 4:**
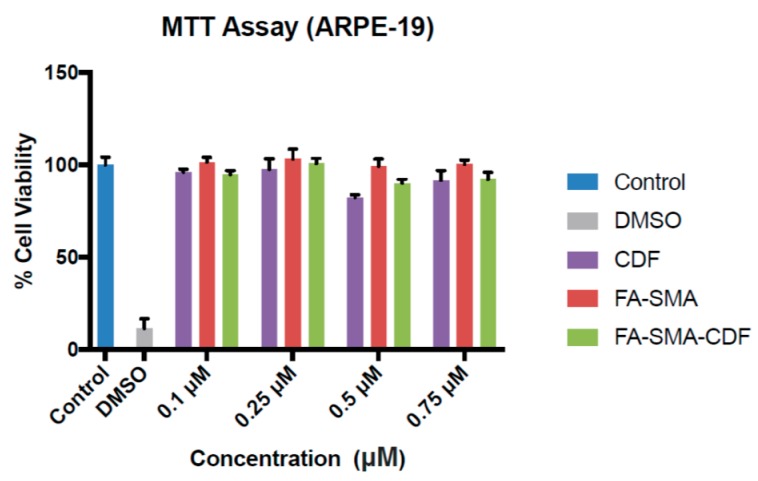
In vitro cell viability assay showing the percentage of cell viability observed at 24 h after treating ARPE-19 cells with the pure drug (CDF), FA-SMA and FA-SMA-CDF. Data are expressed as the percentage of control cells and the mean ± SEM.

**Figure 5:**
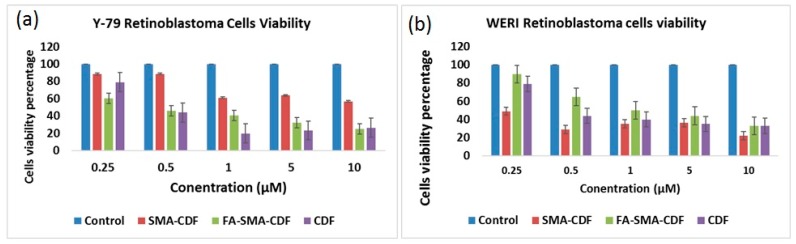
(**a**) In vitro cell viability assay showing the percentage of cell viability observed at 48 h after treating Y-79 cells with various formulations is shown (*n* = 8); (**b**) MTT assay observed at a 48-h treatment of WERI-RB1 cells with SMA-CDF and FA-SMA-CDF as compared to the same treatment is shown (*n* = 8).

The MTT assay was performed to study the cytotoxicity of CDF, FA-SMA and FA-SMA-CDF on normal retinal (ARPE-19) cells. The cytotoxicity of CDF and FA-SMA-CDF at concentrations of 0.1, 0.25, 0.5 and 0.75 μM was tested for 24 h. After 24 h of incubation, none of the tested concentrations of CDF and the formulation (FA-SMA-CDF) showed any cytotoxicity on the normal ARPE-19 cells, except 0.5 µM, which showed some cytotoxicity (Figure 4). Furthermore, the cytotoxicity (MTT assay) of the CDF-loaded nanoformulation was performed against two different types of retinal cancer cell lines (Y-79 and WERI-RB1) to determine the efficacy of the developed formulations against different types of retinoblastoma cells. Plain polymer (FA-SMA) showed very little toxicity to all of the experimental cell lines with a cell viability of more than 93%, which confirmed the safety/biocompatibility of the polymer conjugate. The anticancer activity of FA-SMA-CDF nanomicelles was compared with SMA-CDF and the free drug. The targeted nanomicelles exhibited an improvement in anticancer activity in RB Y-79 cells after incubation at 48 h with respect to the free drug and drug-loaded non-targeted nanomicelles. The result of the cytotoxicity revealed a significant difference in the toxicity of folate-based nanomicelles in comparison to the plain drug, CDF. For instance, the FA-decorated micelles led to a statistically-significant increase (*p* < 0.01) in the cytotoxicity of CDF in the Y-79 cell line (Figure 5a,b).

## 4. Discussion

Retinoblastoma (RB) is a cancer that develops in the retina. Its occurrence is mostly in children below the age of five years. Current therapies comprise radiation therapy, cryotherapy, laser therapy, chemotherapy and enucleation. These therapies have very significant side effects and, if not treated properly, can lead to the disease spreading to both eyes and eventually throughout the body. To overcome the drawbacks associated with current remediations, an alternative non-invasive therapy is urgently needed. Therefore, natural analogs such as CDF that show selectivity to tumor cell killing are some of the promising agents that can be used as an alternative for the treatment of retinoblastoma. However, due to the hydrophobicity of CDF, it is hard to prepare a clear aqueous solution for ophthalmic use. Hence, our aim was to fabricate CDF-loaded micelles using a water-soluble polymer conjugated to folic acid (FA-SMA) to enhance the drug delivery and targeting ability of the micelles to folate-overexpressing retinal tumor cells. In this regard, it is important to note that folate expression is relatively low or sparse in normal retinal cells, as well as normal healthy tissues and organs of the body, making it a selective ligand for targeted drug delivery [12,23].

CDF has been synthesized and tested to be a superior agent in terms of its chemical stability and bioavailability compared to its natural counterpart, curcumin. CDF has been shown to be a very promising anticancer agent with the potential to treat several cancer cells and overcome drug resistance [24,25]. However, photodegradation and the hydrophobicity profile of CDF are some of the primary challenges in dosage preparation and administration. Our previous studies suggested that SMA co-polymeric micelles could improve the aqueous solubility of various hydrophobic compounds, enhance drug accumulation at the tumor site and minimize adverse side effects with increased anticancer activity in vitro and in vivo [26,27,28]. Therefore, the goal of the current study was to design an efficient folate-based targeted nanomicelle system loaded with hydrophobic drug (CDF) that could be useful for targeting retinoblastoma cells. In this regard, the recently-developed fluorocurcumin analog, CDF, has shown improved stability, higher therapeutic potential and a 16-fold increased half-life compared to curcumin [20,21].

FA-SMA conjugates were synthesized and characterized by ^1^H NMR and FTIR spectroscopy (Figure 2a,b). Then, CDF was successfully loaded in non-targeted (SMA-CDF) and targeted (FA-SMA-CDF) nanomicelles, for comparisons. The FA present on FA-SMA is expected to be on the surface of the nanomicelles, with the ability to bind effectively to folate receptors overexpressed on retinoblastoma cells, whereas it does not have any specific ability to bind to normal cells and tissues, thereby helping reduce the side effects.

We analyzed our new delivery system for size and morphology by DLS and TEM. Our results indicated that the nanoformulations were in the ideal nano-size range with a narrow size and polydispersity index (PDI) for retinal cancer cell targeting. The particle size data confirmed that there was a minimum increase in particle size following conjugation with folate (FA-SMA-CDF). Furthermore, our nanomicelles showed a unimodal size distribution, as shown in Figure 3a. Furthermore, TEM images proved the spherical morphology of nanomicelles, and the results indicated a slight increase in the particle size of FA-SMA-CDF compared to SMA-CDF, which is reasonable due to the attachment of the folate ligand as determined by DLS. The free drug, like the parent compound curcumin, suffers from poor aqueous solubility and remains highly prone to photodegradation. Therefore, CDF can lose its anticancer activity dramatically when exposed to light. However, when CDF is encapsulated in the nanomicelles, it is found to be less susceptible to photodegradation. The CDF in FA-SMA and SMA nanomicelles were found to be stable for at least two months under varying ambient and higher temperature conditions (~60 °C) as shown in Table 2.

Human retinoblastoma cell line Y-79 (overexpressing high folate receptors) and WERI-Rb1 (a relatively lower folate receptor expression cell line) were chosen for the in vitro cytotoxicity assay. In vitro cell viability assay (Figure 5a) proved that the targeted formulation FA-SMA-CDF represented higher anticancer activity with a decrease in IC_50_ when tested on Y-79 as compared to non-targeted formulation SMA-CDF. In Y-79 cell lines, IC_50_ values after the 48-h incubation were found to be 1.02 ± 0.55 and 4 ± 1.64 µM for the FA-SMA-CDF targeted formulation and the SMA-CDF non-targeted formulation respectively. The higher anticancer activity of FA decorated nanomicelles FA-SMA-CDF found in Y-79 having overexpression of folate receptors suggested a higher cellular uptake of targeted formulation FA-SMA-CDF in these cell lines as compared to the non-targeted SMA-CDF formulation. In this case, the folate receptor-mediated endocytosis (Figure 1) may be the primary mechanism for faster and better internalization of nanomicelles into RB cells followed by efficient CDF release within the cells. On the other hand, the results observed in WERI-RB1 cell lines did not reveal any significant difference between the targeted and non-targeted formulations. The observed results may be attributed to low folate expression in WERI-RB1 cells as compared to Y-79 cells. More thorough evaluation may be needed to ascertain the observed findings. However, it can be clearly stated that the targeted formulations showed enhanced cytotoxicity to the folate-overexpressing Y-79 cells, which is a promising finding for treating folate-overexpressing retinoblastoma cells. Indeed, the results of our study justified the promise of targeted nanomicelles (FA-SMA-CDF) in not only enhancing CDF′s poorly aqueous solubility, but also improving cellular uptake with better activity in Y-79 RB cells. Furthermore, our results also suggested the safety profile of FA-SMA polymer, as well as FA-SMA-CDF, with almost no reduction in cell viability in the normal retinal cell lines. Taken together, our results portend promising potential for the future translation of nanoformulations for ocular cancers.

## 5. Conclusions

FA-conjugated copolymer SMA exhibited a promising avenue for targeting the delivery of CDF, a potent cytotoxic anticancer analog of curcumin. The FA-decorated nanomicelles have excellent properties, such as improved drug stability and high drug loading with a sustained release profile, enhancing delivery of a high dose of CDF to cancer cells overexpressing the folate receptors (via receptor-mediated endocytosis). Furthermore, the targeted and non-targeted formulations could increase the cytotoxicity of retinoblastoma cells while demonstrating lower cytotoxicity on cells of normal ocular origin, indicating their safety on the normal cells and healthy tissues. The results suggest a promising future for FA-SMA-CDF and SMA-CDF nanomicelles for the treatment of retinal cancers in the clinic.

## Figures and Tables

**Table 1:** Characterization of FA-SMA-CDF micelles. EE, encapsulation efficiency.

Sample	Hydrodynamic size (nm)	PDI	Zeta potential (mV)	EE (%)
FA-SMA-CDF	193.6 ± 20 nm	0.175 ± 0.05	–7.12 ± 4	75.98 ± 12
SMA-CDF	183 ± 31 nm	0.183 ± 0.07	–34 ± 5	70.21 ± 9

Abbreviations: SMA, styrene maleic acid; FA, Folic Acid; PDI, polydispersity index.

**Table 2:** Percent drug content of nanomicellar formulations after sixty days (*n* = 3). Values expressed as the mean ± standard deviation (SD).

Formulation	Temperature		
	4 °C	25 °C	35 °C
FA-SMA-CDF (% drug content recovery)	100.73 ± 2.10	96.9 ± 3.2	89.76 ± 2.76
SMA-CDF (% drug content recovery)	99.41 ± 1.83	98.42 ± 1.81	90.91 ± 1.54

## References

[1] Sreenivasan S., Thirumalai K., Danda R., Krishnakumar S. (2012). Effect of curcumin on miRNA expression in human Y79 retinoblastoma cells. Curr. Eye Res..

[2] Di Fiore R., Drago-Ferrante R., D’Anneo A., Augello G., Carlisi D., De Blasio A., Giuliano M., Tesoriere G., Vento R. (2013). In human retinoblastoma Y79 cells okadaic acid-parthenolide co-treatment induces synergistic apoptotic effects, with PTEN as a key player. Cancer Biol. Ther..

[3] Aerts I., Pacquement H., Doz F., Mosseri V., Desjardins L., Sastre X., Michon J., Rodriguez J., Schlienger P., Zucker J.M. (2004). Outcome of second malignancies after retinoblastoma: A retrospective analysis of 25 patients treated at the Institut Curie. Eur. J. Cancer.

[4] Boice J.D., Abramson D.H. (1998). Cancer incidence after retinoblastoma: Radiation dose and sarcoma risk. Surv. Ophthalmol..

[5] Zhang J., Benavente C.A., McEvoy J., Flores-Otero J., Ding L., Chen X., Ulyanov A., Wu G., Wilson M., Wang J. (2012). A novel retinoblastoma therapy from genomic and epigenetic analyses. Nature.

[6] Luong D., Kesharwani P., Killinger B.A., Moszczynska A., Sarkar F.H., Padhye S., Rishi A.K., Iyer A.K. (2016). Solubility enhancement and targeted delivery of a potent anticancer flavonoid analogue to cancer cells using ligand decorated dendrimer nano-architectures. J. Colloid Interface Sci..

[7] Anand P., Kunnumakkara A., Newman R. (2007). Bioavailability of curcumin: Problems and promises. Pharmaceutics.

[8] Padhye S., Banerjee S., Chavan D., Pandye S. (2009). Fluorocurcumins as cyclooxygenase-2 inhibitor: Molecular docking, pharmacokinetics and tissue distribution in mice. Pharmaceutical.

[9] Padhye S., Yang H., Jamadar A., Cui Q. (2009). New difluoro Knoevenagel condensates of curcumin, their Schiff bases and copper complexes as proteasome inhibitors and apoptosis inducers in cancer cells. Pharmaceutical.

[10] Kanwar S.S., Yu Y., Nautiyal J., Patel B.B., Padhye S., Sarkar F.H., Majumdar A.P. (2011). Difluorinated-curcumin (CDF): A novel curcumin analog is a potent inhibitor of colon cancer stem-like cells. Pharm. Res.

[11] Allison E., Rizzuti B.A., Ira J., Dunkel M.D.H.A. (2008). The adverse events of chemotherapy for retinoblastoma. Arch. Ophthalmol..

[12] Boddu S.H.S., Jwala J., Chowdhury M.R., Mitra A.K. (2010). In vitro evaluation of a targeted and sustained release system for retinoblastoma cells using Doxorubicin as a model drug. J. Ocul. Pharmacol. Ther..

[13] Amaral L., Engi H., Viveiros M., Molnar J. (2007). Comparison of multidrug resistant efflux pumps of cancer and bacterial cells with respect to the same inhibitory agents. In Vivo.

[14] Yoshihiko E. (1997). RB protein status and chemosensitivity in non-small cell lung cancers. Oncology.

[15] Jwala J., Vadlapatla R.K., Vadlapudi A.D., Boddu S.H.S., Pal D., Mitra A.K. (2012). Differential expression of folate receptor-alpha, sodium-dependent multivitamin transporter, and amino acid transporter (B (0, +)) in human retinoblastoma (Y-79) and retinal pigment epithelial (ARPE-19) cell lines. J. Ocul. Pharmacol. Ther..

[16] Kansara V., Pal D., Jain R. (2005). Identification and functional characterization of riboflavin transporter in human-derived retinoblastoma cell line (Y-79): Mechanisms of cellular uptake and translocation. J. Ocul..

[17] Yoo H.S., Park T.G. (2004). Folate receptor targeted biodegradable polymeric doxorubicin micelles. J. Control Release.

[18] Shiokawa T., Hattori Y., Kawano K., Ohguchi Y. (2005). Effect of polyethylene glycol linker chain length of folate-linked microemulsions loading aclacinomycin A on targeting ability and antitumor effect in vitro and in vivo. Clin. Cancer.

[19] Kansara V., Paturi D., Luo S., Gaudana R. (2008). Folic acid transport via high affinity carrier mediated system in human retinoblastoma cells. Int. J..

[20] Kesharwani P., Banerjee S., Padhye S., Sarkar F.H., Iyer A.K. (2015). Parenterally administrable nano-micelles of 3, 4-difluorobenzylidene curcumin for treating pancreatic cancers. Colloid Surf. B Biointer..

[21] Kesharwani P., Banerjee S., Padhye S., Sarkar F.H., Iyer A.K. (2015). Hyaluronic acid engineered nanomicelles loaded with 3,4-difluorobenzylidene curcumin for targeted killing of CD44^+^ stem-like pancreatic cancer cells. Biomacromolecules.

[22] Harmonisation I.C. Validation of analytical procedures: Text and methodology. ICH Harmonised Tripartite Guideline, Proceedings of the International Conference on Harmonisation of Technical Requirements for Registration of Pharmaceuticals for Human Use.

[23] Yoo H.S., Park T.G. (2004). Folate-receptor-targeted delivery of doxorubicin nano-aggregates stabilized by doxorubicin-PEG-folate conjugate. J. Control. Release.

[24] Luong D., Sau S., Kesharwani P., Iyer A.K. (2017). Polyvalent folate-dendrimer-coated iron oxide theranostic nanoparticles for simultaneous magnetic resonance imaging and precise cancer cell targeting. Biomacromolecules.

[25] Gawde K.A., Kesharwani P., Sau S., Sarkar F.H., Padhye S., Kashaw S.K., Iyer A.K. (2017). Synthesis and characterization of folate decorated albumin bio-conjugate nanoparticles loaded with a synthetic curcumin difluorinated analogue. J. Colloid Interface Sci..

[26] Kesharwani P., Jain K., Jain N.K. (2014). Dendrimer as nanocarrier for drug delivery. Prog. Polym. Sci..

[27] Kesharwani P., Banerjee S., Gupta U., Mohd Amin M.C.I., Padhye S., Sarkar F.H., Iyer A.K. (2015). PAMAM dendrimers as promising nanocarriers for RNAi therapeutics. Mater. Today.

[28] Kesharwani P., Xie L., Banerjee S., Mao G., Padhye S., Sarkar F.H., Iyer A.K. (2015). Hyaluronic acid-conjugated polyamidoamine dendrimers for targeted delivery of 3,4-difluorobenzylidene curcumin to CD44 overexpressing pancreatic cancer cells. Colloid. Surf. B. Biointer..

